# Supplementary figures and images for: So you think you can PLS-DA?
Source: BMC Bioinformatics. 2020 Dec 9;21(Suppl 1):2. doi: 10.1186/s12859-019-3310-7 (PMC7724830; doi:10.1186/s12859-019-3310-7)

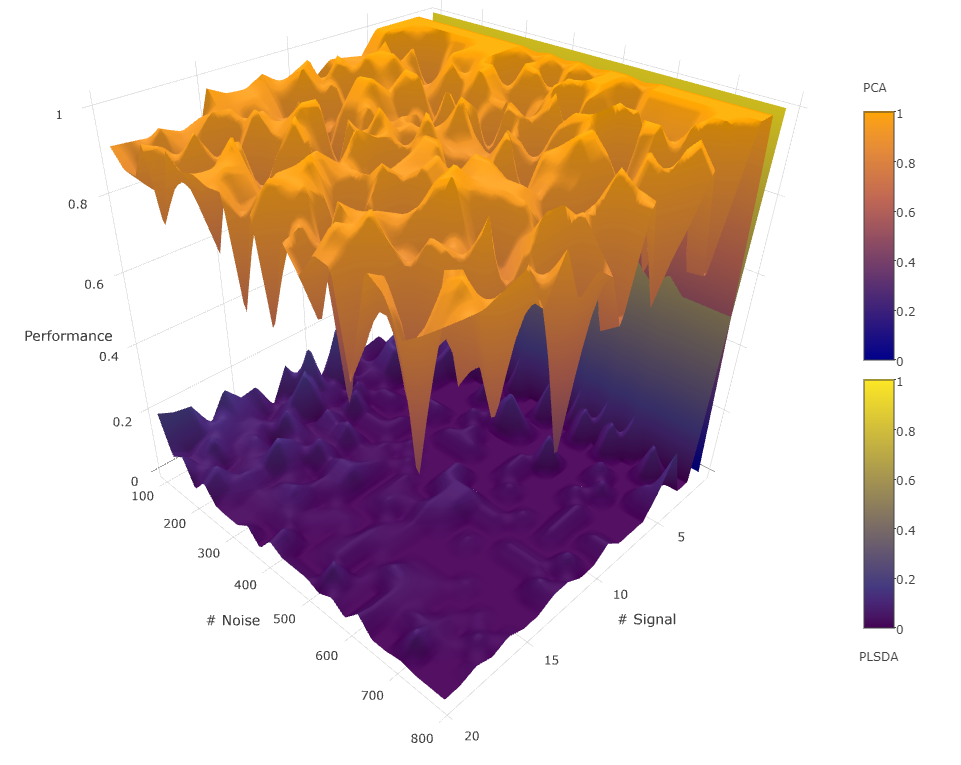

Supplement: Supplementary file 1 — Additional file 1 Figure S1. Performance for linearly separable points model, varying signal and noise. [file 12859_2019_3310_MOESM1_ESM.png]

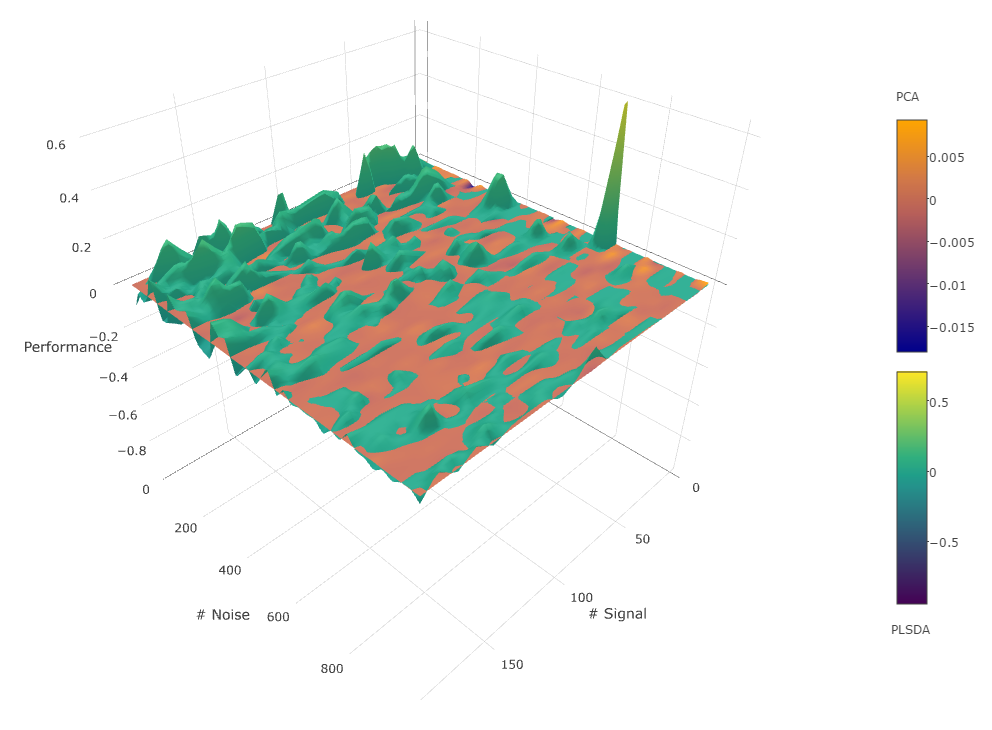

Supplement: Supplementary file 2 — Additional file 2 Figure S2. Performance for linearly separable points model with the cosine model, varying signal and noise. [file 12859_2019_3310_MOESM2_ESM.png]

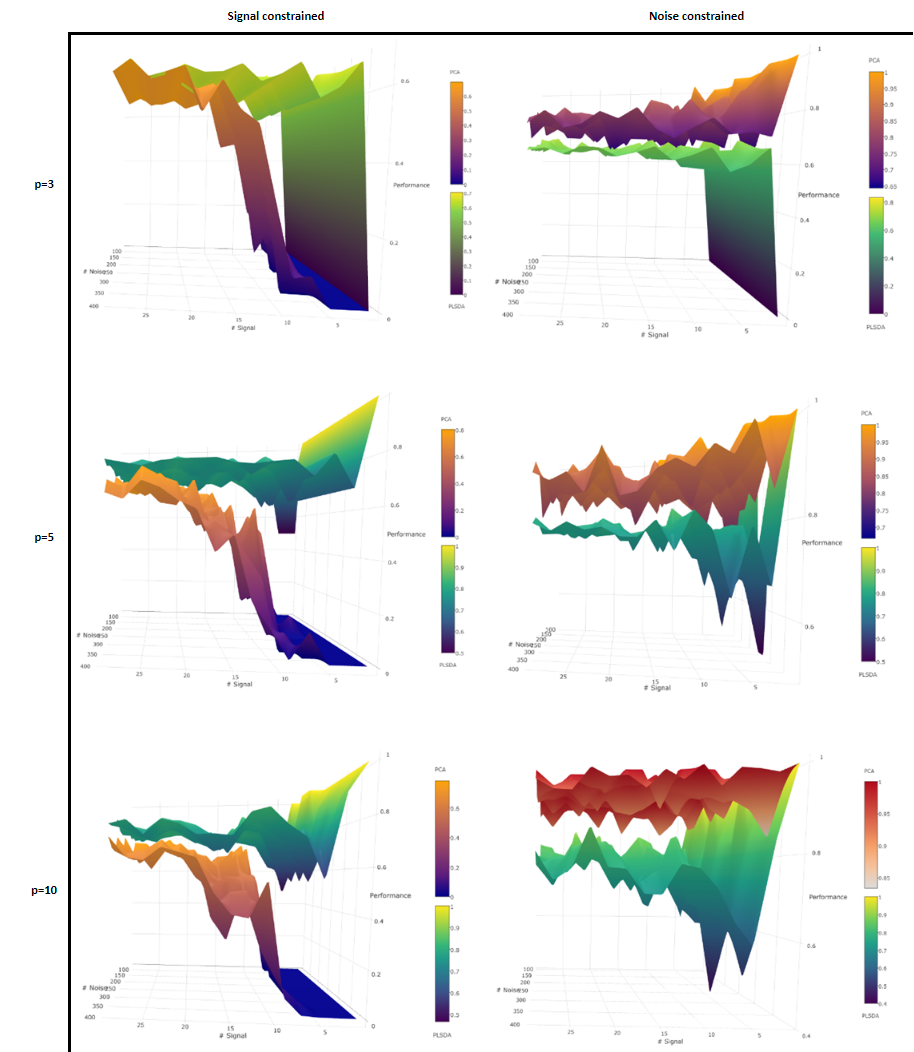

Supplement: Supplementary file 3 — Additional file 3 Figure S3. Performance table for different configurations of the interval model. [file 12859_2019_3310_MOESM3_ESM.png]

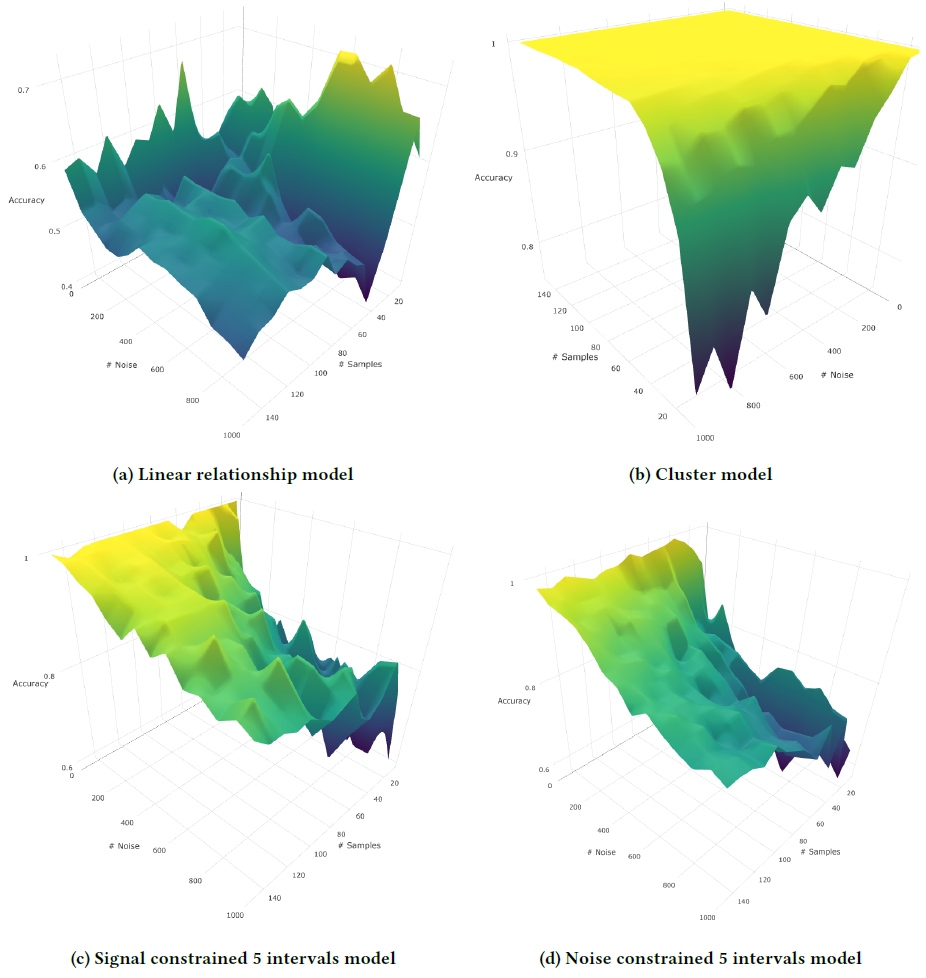

Supplement: Supplementary file 4 — Additional file 4 Figure S4. Classification accuracy for the different data models. [file 12859_2019_3310_MOESM4_ESM.png]

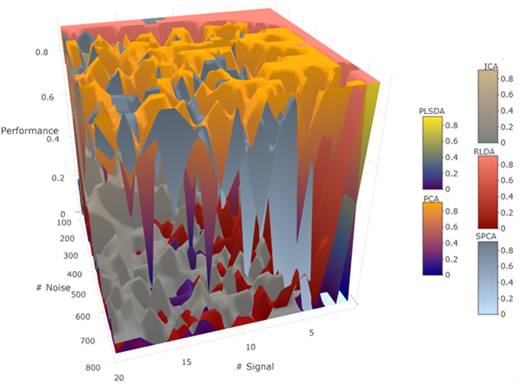

Supplement: Supplementary file 5 — Additional file 5 Figure S5. Performance for linearly separable points model, varying signal and noise. [file 12859_2019_3310_MOESM5_ESM.jpg]

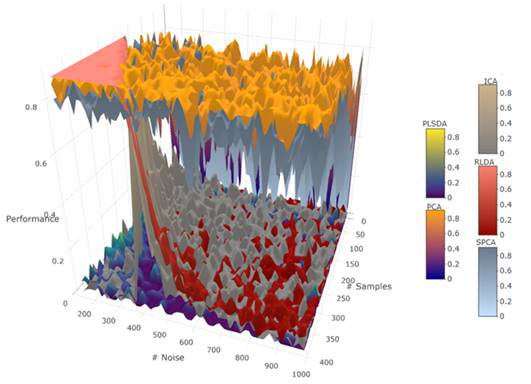

Supplement: Supplementary file 6 — Additional file 6 Figure S6. Performance for linearly separable points model, varying samples and noise. [file 12859_2019_3310_MOESM6_ESM.jpg]

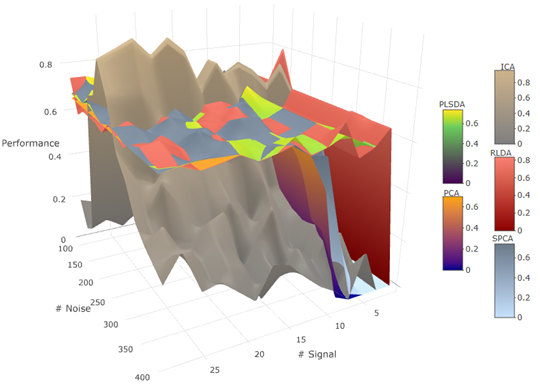

Supplement: Supplementary file 7 — Additional file 7 Figure S7. Performance of other methods, signal constrained interval with p=3. [file 12859_2019_3310_MOESM7_ESM.png]

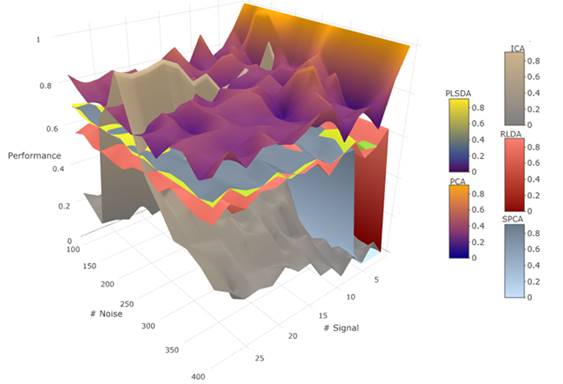

Supplement: Supplementary file 8 — Additional file 8 Figure S8. Performance of other methods, noise constrained interval with p=3. [file 12859_2019_3310_MOESM8_ESM.jpg]

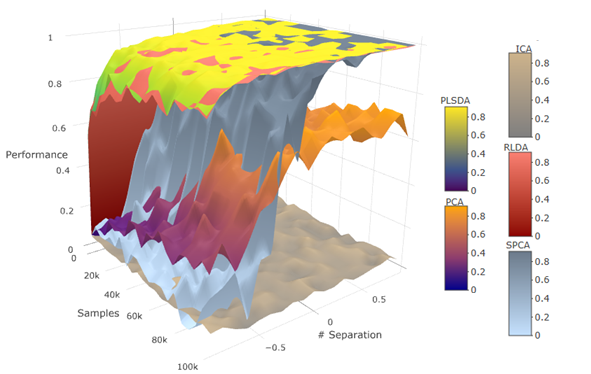

Supplement: Supplementary file 9 — Additional file 9 Figure S9. Performance of other methods for the cluster model, High number of samples. [file 12859_2019_3310_MOESM9_ESM.png]
